# Higher ARNT expression is associated with the sparsely granulated phenotype in somatotroph pituitary adenomas: An immunohistochemical study

**DOI:** 10.1007/s11102-026-01776-5

**Published:** 2026-10-16

**Authors:** Cristiana Călin, Iulia Florentina Burcea, Ramona Dobre, Roxana-Ioana Dumitriu Stan, Mihai Alin Publik, Dana Cristina Terzea, Mihail-Constantin Ceaușu, Cătălina Poiană

**Affiliations:** 1 Carol Davila University of Medicine and Pharmacy, Bucharest, 020021 Romania; 2 Department of Endocrinology, ”C.I. Parhon” National Institute of Endocrinology, Bucharest, 011863 Romania; 3 Department of Pathology, ”C.I. Parhon” National Institute of Endocrinology, Bucharest, 011863 Romania

**Keywords:** Acromegaly, Somatotroph pituitary adenoma, Aryl hydrocarbon receptor nuclear translocator (ARNT), Aryl hydrocarbon receptor-interacting protein (AIP), Aryl hydrocarbon receptor (AHR), Cytokeratin pattern

## Abstract

**Purpose:**

Somatotroph pituitary adenomas are a heterogeneous group comprising distinct histopathological subtypes, including the well-defined densely granulated (DG) and sparsely granulated (SG) phenotypes. While the aryl hydrocarbon receptor-interacting protein (AIP) pathway has been implicated in pituitary tumorigenesis, the role of aryl hydrocarbon receptor nuclear translocator (ARNT) in somatotroph tumors remains poorly characterized.

**Methods:**

We retrospectively analyzed 71 surgically treated somatotroph pituitary adenomas, clinically managed in a tertiary endocrinology center between 2020 and 2025. Immunohistochemical expression of AIP, AHR and ARNT was evaluated and correlated with clinicopathological characteristics, including cytokeratin pattern, hormonal expression and Ki-67 index.

**Results:**

Moderate/strong ARNT expression was more frequent in SG than in DG adenomas (60% vs. 17.6%, *p* = 0.002). Patients with moderate/strong ARNT expression were younger than those with absent/weak expression (median 39 vs. 46 years, *p* = 0.044). In multivariable analysis, ARNT expression remained associated with the SG phenotype after adjustment for age and sex (adjusted OR 6.84, 95% CI 2.05–22.84; *p* = 0.002). No significant associations were observed between ARNT expression and Ki-67 index, baseline GH or IGF1 levels, AIP/AHR immunoexpression or the evaluated clinical outcomes, including biochemical control during first-generation somatostatin receptor ligand therapy.

**Conclusions:**

Increased ARNT expression is associated with the sparsely granulated phenotype in somatotroph pituitary adenomas, suggesting that ARNT expression may reflect tumor differentiation rather than proliferative activity. However, the present findings do not demonstrate additional prognostic or therapeutic utility beyond granulation pattern. Further molecular and transcriptomic studies are warranted to clarify the biological role of ARNT.

**Supplementary Information:**

The online version contains supplementary material available at https://doi.org/10.1007/s11102-026-01776-5.

## Introduction

 Acromegaly is most often caused by a GH-secreting pituitary adenoma and remains associated with substantial cardiovascular, metabolic, respiratory, skeletal and neoplastic morbidity despite therapeutic advances [1]. Within the current World Health Organization classification, these lesions are designated as pituitary neuroendocrine tumors (PitNETs). Nevertheless, the traditional term pituitary adenoma remains widely employed in clinical practice and the medical literature, reflecting its longstanding use and continued familiarity among clinicians [2, 3]. Differences in lineage maturation, invasion and treatment responsiveness have made histological subtype increasingly relevant to integrated prognostic assessment [2, 3]. Personalized management therefore depends on biomarkers that capture this biological diversity at diagnosis and after surgery [1, 3].

Among somatotroph adenomas, cytokeratin-defined densely granulated (DG) and sparsely granulated (SG) subtypes are the most reproducible clinicopathological categories. Across multiple series, SG tumors have been associated with younger age at diagnosis, larger size, greater invasiveness, lower SSTR2 expression and a less favorable response to first-generation somatostatin receptor ligands, whereas DG tumors more often behave as treatment-sensitive, more differentiated lesions [4–7]. Such differences are clinically relevant because they influence both post-operative remission and subsequent medical treatment choices. Nevertheless, these associations are not entirely uniform across cohorts, indicating that additional biomarkers could refine subtype-based risk stratification [4–7].

The aryl hydrocarbon receptor-interacting protein (AIP), aryl hydrocarbon receptor (AHR), and aryl hydrocarbon receptor nuclear translocator (ARNT) constitute an interconnected molecular network. Germline *AIP* pathogenic variants established *AIP* as a pituitary tumor-predisposition gene, particularly in GH-secreting disease [8]. Somatotroph adenomas in patients with germline AIP pathogenic variants are more frequently sparsely granulated [9]. In pituitary tumors, altered AHR and AIP expression has also been documented, supporting relevance of this signaling network beyond hereditary cases [10]. ARNT (also termed HIF-1β) is the obligate dimerization partner for both ligand-activated AHR and HIF1-α transcription factors, placing it at the intersection of xenobiotic signaling, hypoxia and cell-differentiation programs [11]. Notably, reduced ARNT expression has been reported in *AIP*-mutant pituitary adenomas, and Aip-deficient mice develop GH-predominant pituitary tumors with aberrant ARNT/ARNT2 expression, suggesting disturbed ARNT-dependent signaling in pituitary tumorigenesis [12, 13].

However, most available pituitary evidence on ARNT comes from AIP-linked human tumors or experimental models rather than subtype-focused analyses of somatotroph adenomas [12, 13]. Because granulation pattern captures both tumor differentiation and treatment response, examining ARNT expression in a clinicopathologically characterized cohort of surgically treated patients with somatotroph pituitary adenomas may clarify whether this molecule is associated with a distinct somatotroph phenotype.

## Materials and methods

### Patients and study design

We retrospectively analyzed 71 patients with surgically treated somatotroph pituitary adenomas, clinically managed at a tertiary endocrinology center between January 2020 and December 2025. The diagnosis of acromegaly was established based on characteristic clinical features, elevated age-adjusted insulin-like growth factor 1 (IGF1) levels, and inadequate growth hormone (GH) suppression during oral glucose tolerance testing (OGTT) when performed, in accordance with international guideline recommendations [14] and the Romanian national therapeutic protocol for acromegaly (H005E) applicable during the study period [15]. Baseline GH concentrations were available for all patients and were used for the biochemical analyses in the present study.

Only patients with available formalin-fixed, paraffin-embedded (FFPE) tumor tissue were included in the study. AIP, AHR, and ARNT immunohistochemistry was performed specifically for the purposes of the present study on archived FFPE tumor tissue. Clinical, biochemical, radiological, and histopathological data were retrospectively collected from medical records. None of the patients included in the study had a known family history suggestive of familial isolated pituitary adenoma (FIPA). Germline AIP testing was not systematically performed across the entire cohort. Among patients for whom genetic testing was available, no pathogenic or likely pathogenic AIP variants or variants of uncertain significance (VUS) were identified according to ACMG/AMP criteria [16].

Tumors were classified according to the World Health Organization classification of pituitary tumors. Granulation pattern was evaluated using AE1/AE3 immunohistochemistry and tumors were classified as densely granulated (DG) or sparsely granulated (SG) somatotroph adenomas according to established histopathological criteria [2].

The study was conducted in accordance with the Declaration of Helsinki and approved by the Institutional Ethics Committee of “C.I. Parhon” National Institute of Endocrinology, Bucharest, Romania (Ethics Approval No. 25/19.07.2023).

### Clinical and radiological evaluation

Serum GH and IGF1 concentrations were measured using chemiluminescent immunoassays (CLIA) on the LIAISON^®^ XL analyzer (DiaSorin S.p.A., Saluggia, Italy). Age at diagnosis, sex, baseline GH levels and IGF1 levels expressed as times the upper limit of normal (×ULN) were recorded.

Tumor size was evaluated on magnetic resonance imaging (MRI), and lesions were classified as microadenomas (< 10 mm) or macroadenomas (≥ 10 mm). Tumors were considered invasive in the presence of Knosp grade 3 or 4 according to the original Knosp classification [17] and/or sphenoid sinus invasion on preoperative MRI.

Postoperative biochemical remission was determined from the postoperative endocrine assessment documented in the medical records, generally performed 3–6 months after surgery, and was defined by normalization of age-adjusted serum IGF1. Patients without postoperative biochemical remission were considered to have persistent disease. Residual tumor was defined as radiologically identifiable residual adenoma on postoperative pituitary MRI. Among patients with persistent disease after surgery who subsequently received medical therapy, biochemical disease control at the last evaluation was defined by normalization of age-adjusted serum IGF1. Biochemical response to first-generation somatostatin receptor ligands (octreotide LAR or lanreotide Autogel) was assessed according to IGF1 control; normalization of age-adjusted serum IGF1 was considered biochemical control, whereas persistently elevated IGF1 indicated inadequate biochemical control during first-generation SRL therapy, in accordance with current consensus recommendations [18].

Preoperative medical treatment was recorded, including exposure to somatostatin receptor ligands (SRLs) and treatment duration. Patients who had not received medical therapy for acromegaly before surgery were considered treatment-naive.

### Histopathological and immunohistochemical evaluation

All surgical specimens were rapidly fixed in ready-to-use 10% neutral buffered formalin (4% formaldehyde solution; Diapath, Martinengo, Italy; F0047) and embedded in paraffin according to standard histopathological protocols. Serial 3-µm sections were stained with hematoxylin and eosin (H&E) for routine microscopic evaluation and confirmation of the histopathological diagnosis. Additional serial sections were used for immunohistochemical studies.

Immunohistochemical staining was performed using the BOND-MAX Fully Automated IHC and ISH Staining System (Leica Microsystems, Nussloch, Germany), with BOND reagents according to the manufacturer’s standard protocol. The automated immunohistochemistry protocol consisted of deparaffinization followed by heat-induced epitope retrieval using BOND Epitope Retrieval Solution 1 (Leica Microsystems, Deer Park, IL, USA; AR9961) for 20 min at 100 °C. The incubation time for all primary antibodies was 15 min. Detection was performed using the BOND Polymer Refine Detection System (Leica Microsystems, Deer Park, IL, USA; DS9800). Briefly, the protocol consisted of peroxide blocking for 5 min, primary antibody incubation for 15 min, post-primary reagent incubation for 8 min, polymer reagent incubation for 8 min, Mixed DAB Refine for 10 min, followed by hematoxylin counterstaining for 5 min. Representative photomicrographs were acquired using an Olympus CX43 upright microscope (Evident Scientific, Tokyo, Japan; CX43) equipped with an Olympus SC180 digital microscope camera (Evident Scientific, Tokyo, Japan; SC180) and a ×40 objective, using cellSens Entry microscope imaging software (Evident Scientific, Tokyo, Japan).

Immunohistochemical characterization included pituitary hormones (GH, PRL, TSH), PIT1, Ki-67, AE1/AE3 cytokeratin, AIP, AHR and ARNT. Antibody specifications and dilutions are summarized in Table 1.

**Table 1:** Primary antibodies used for immunohistochemical evaluation

Antibody	Manufacturer	Reference number	Clone	Host species	Localization	Dilution	RRID
GH	Abcam, Cambridge, UK	ab268066	GH-3155	Mouse monoclonal	Cytoplasmic	1:100	AB_3756726
PRL	Abcam, Cambridge, UK	ab183967	EPR19386	Rabbit monoclonal	Cytoplasmic	1:100	AB_2814839
TSH-ß	Abcam, Cambridge, UK	ab155958	EPR8198	Rabbit monoclonal	Cytoplasmic	1:250	AB_2928117
PIT1	Abcam, Cambridge, UK	ab313642	EPR28191-52	Rabbit monoclonal	Nuclear	1:100	AB_3756727
Ki-67	Abcam, Cambridge, UK	ab16667	SP6	Rabbit monoclonal	Nuclear	1:200	AB_302459
AE1/AE3	Abcam, Cambridge, UK	ab86734	AE1/AE3 + 5D3	Mouse monoclonal	Cytoplasmic	1:250	AB_10674321
AIP	Abcam, Cambridge, UK	ab192024	EPR13585	Rabbit monoclonal	Cytoplasmic	1:100	AB_3756728
AHR	Sigma-Aldrich, St. Louis, MO, USA	HPA029723	Polyclonal	Rabbit polyclonal	Cytoplasmic	1:100	AB_10599025
ARNT (HIF-1β)	Sigma-Aldrich, St. Louis, MO, USA	WH0000405M1	3D10	Mouse monoclonal	Nuclear	1:300	AB_1839842

Abbreviations: *GH* growth hormone, *PRL* prolactin, *TSH* thyroid-stimulating hormone, *PIT1* pituitary-specific positive transcription factor 1; Ki-67, proliferation marker Ki-67; AE1/AE3, pancytokeratin marker, *AIP* aryl hydrocarbon receptor-interacting protein, *AHR* aryl hydrocarbon receptor, *ARNT* aryl hydrocarbon receptor nuclear translocator, *RRID* Research Resource Identifier

For ARNT immunohistochemistry, a mouse monoclonal anti-ARNT antibody (clone 3D10; Sigma-Aldrich, WH0000405M1; corresponding to Abnova H00000405-M01) was selected based on its documented reactivity with human ARNT and manufacturer validation for immunohistochemistry in formalin-fixed, paraffin-embedded tissue. The antibody was raised against a recombinant N-terminal fragment of human ARNT (amino acids 1-110). Manufacturer validation includes immunoperoxidase staining of ARNT in formalin-fixed, paraffin-embedded human lung tissue.

For AIP, AHR, and ARNT immunohistochemistry, appropriate positive and negative controls were included. Normal adenohypophyseal tissue was used as a positive control for AIP. For AHR, positive immunoreactivity was confirmed in lymphocytes within secondary lymphoid aggregates in liver tissue, whereas formalin-fixed, paraffin-embedded normal human lung tissue was used as a positive control for ARNT. Negative controls were performed for each antibody by replacing the primary antibody with antibody diluent alone. Antibody working dilutions were optimized starting from the manufacturer-recommended dilutions; where necessary, dilution trials were performed, and staining intensity, specificity, and background were assessed by the pathologists before evaluation of the study cohort. Representative images of the positive and negative controls for AIP, AHR, and ARNT immunohistochemistry are provided in Supplementary Figure S1.

Hormonal markers together with AIP and AHR were evaluated according to cytoplasmic staining patterns, whereas ARNT, Ki-67 and PIT1 expression were assessed at the nuclear level. Somatotroph lineage was established based on GH immunoreactivity and PIT1 expression, in conjunction with the overall immunohistochemical profile. PRL immunoreactivity was also assessed in all tumors. All tumors included in the study were classified as somatotroph adenomas. Granulation pattern was assessed using AE1/AE3 cytokeratin immunostaining. Tumors showing a predominantly perinuclear cytokeratin staining pattern, with absent or only focal fibrous bodies, were classified as DG, whereas tumors showing fibrous bodies in > 70% of tumor cells were classified as SG, according to established morphological criteria [2]. No tumors with an intermediate or mixed cytokeratin pattern were identified in the study cohort.

Hormonal immunoreactivity was semi-quantitatively graded according to the percentage of positive tumor cells across the entire tissue section, using a four-tier scoring system: score 0 (0–10%), score 1 (10–30%), score 2 (30–60%), and score 3 (> 60%). Immunohistochemical expression of AIP, AHR and ARNT was assessed semi-quantitatively according to staining intensity, using a four-tier scale: 0, absent; 1, weak; 2, moderate; and 3, strong staining. This approach was based on previously published semi-quantitative immunohistochemical assessments of AIP/AHR and ARNT expression in pituitary adenomas [10, 12]. Granulation pattern had been established by AE1/AE3 immunostaining before AIP, AHR and ARNT scoring; therefore, the pathologists were not blinded to the DG/SG classification.

For the main comparative analyses, ARNT expression was categorized as absent/weak (scores 0–1) or moderate/strong (scores 2–3), according to staining intensity. To assess whether the observed associations were dependent on this dichotomization, the original four-tier ordinal ARNT score (0–3) was additionally evaluated in sensitivity analyses.

The Ki-67 labeling index was expressed as the percentage of tumor cells showing positive nuclear staining. A Ki-67 cut-off of 3% was used based on its established use as a proliferative marker in pituitary tumors and its continued recognition in the revised European Society of Endocrinology Clinical Practice Guideline for aggressive pituitary tumors and pituitary carcinomas [19].

All immunohistochemical slides were independently evaluated by two experienced pathologists with expertise in pituitary pathology who were blinded to clinical outcomes. In cases of discordant interpretation, the slides were jointly reviewed and a consensus diagnosis was established.

### Statistical analysis

Statistical analysis was performed using IBM SPSS Statistics version 30.0 (IBM Corp., Armonk, NY, USA). Continuous variables were expressed as median and interquartile range (IQR), while categorical variables were reported as absolute numbers and percentages.

Comparisons between groups were performed using the Mann-Whitney U test for continuous variables and the chi-square test or Fisher’s exact test for categorical variables, as appropriate. ARNT expression was analyzed both as an ordinal variable (scores 0-3) and as a dichotomized variable (absent/weak expression: scores 0-1; moderate/strong expression: scores 2-3).

Pairwise correlations between AIP, AHR and ARNT immunohistochemical expression were evaluated using Spearman’s rank correlation coefficient (Spearman’s rho), both in the overall cohort and after stratification according to granulation pattern.

Following univariable analyses, an exploratory multivariable logistic regression model was constructed to assess whether the association between moderate/strong ARNT expression and the SG phenotype remained significant after adjustment for age at diagnosis and sex. Variables included in the multivariable model were selected a priori based on biological relevance. Adjusted odds ratios (ORs) with 95% confidence intervals (CIs) were calculated.

A two-sided p-value <0.05 was considered statistically significant.

## Results

### Clinicopathological characteristics of the cohort

A total of 71 patients with surgically treated somatotroph pituitary adenomas were included in the study.

The median age at diagnosis was 46 years (IQR 36.5–54), and most patients were female (63.4%). The majority of tumors were macroadenomas at presentation (64/71, 90.1%). Overall, 27/71 tumors (38.0%) were invasive. Sphenoid sinus invasion was present in 7/71 cases (9.9%). Suprasellar extension was observed in 26/71 tumors (36.6%), and clinical optochiasmatic involvement was documented in 19/71 patients (26.8%).

Median baseline GH level was 9.5 ng/mL (IQR 4.2–19.7), whereas median IGF1 level was 3.4 × ULN (IQR 2.5–4.3), consistent with active acromegaly at diagnosis.

Most patients (69/71, 97.2%) were treatment-naive at the time of surgery. Only two patients (2.8%) had received preoperative first-generation somatostatin receptor ligand therapy: one received octreotide LAR 30 mg every 28 days for 3 years, while the other received lanreotide prolonged release for 6 months as part of a clinical trial.

Histopathological evaluation identified 50 densely granulated (70.4%) and 21 sparsely granulated (29.6%) somatotroph adenomas. Ki-67 labeling index was below 3% in most tumors (73.2%).

Detailed clinicopathological characteristics are presented in Table 2.

**Table 2:** Clinicopathological characteristics of the study cohort

Variable	Total cohort (*n* = 71)
Age at diagnosis, years	46 (36.5–54)
Female sex, n (%)	45 (63.4)
Baseline GH, ng/mL	9.5 (4.2–19.7)
IGF1 xULN	3.4 (2.5–4.3)
Treatment-naive before surgery, n (%)	69 (97.2)
Macroadenoma, n (%)	64 (90.1)
Invasive tumors, n (%)	27 (38)
Sphenoid sinus invasion, n (%)	7/71 (9.9)
Suprasellar extension, n (%)	26/71 (36.6)
Optochiasmatic involvement, n (%)	19/71 (26.8)
Ki-67 < 3%, n (%)	52 (73.2)
Densely granulated tumors, n (%)	50 (70.4)
Sparsely granulated tumors, n (%)	21 (29.6)

Data are presented as median (interquartile range) or number (%)

### Immunohistochemical profile of somatotroph pituitary adenomas

Immunohistochemical findings are summarized in Table 3. Most tumors demonstrated strong GH expression, consistent with somatotroph differentiation, while PIT1 positivity was observed in all cases. PRL immunoreactivity was absent in 45/71 tumors (63.4%) and present to varying degrees in 26/71 tumors (36.6%). In none of the included tumors did PRL immunohistochemical expression exceed GH expression. Cytokeratin staining identified both DG and SG somatotroph adenomas within the cohort

AIP expression was generally preserved, whereas AHR immunoreactivity was absent or weak (scores 0–1) in 69 of 71 tumors (97.2%). ARNT immunoreactivity ranged from absent to strong nuclear expression. For comparative analyses, tumors were subsequently categorized as showing absent/weak (scores 0–1) or moderate/strong (scores 2–3) ARNT expression

**Table 3:** Distribution of immunohistochemical expression scores in the study cohort

Score	GH *n* (%)	AIP *n* (%)	AHR *n* (%)	ARNT *n* (%)
0	0 (0%)	0 (0%)	49 (69%)	21 (29.6%)
1	3 (4.2%)	1 (1.4%)	20 (28.2%)	30 (42.3%)
2	8 (11.3%)	4 (5.6%)	2 (2.8%)	19 (26.8%)
3	60 (84.5%)	66 (93%)	0 (0%)	1 (1.4%)

Data are presented as number (%). GH immunoreactivity was scored according to the percentage of positive tumor cells, whereas AIP, AHR and ARNT were scored according to staining intensity

### Clinicopathological characteristics according to ARNT expression

Clinicopathological characteristics according to ARNT expression are presented in Table 4

In univariable analyses, tumors with moderate/strong ARNT expression (scores 2–3) were more frequently associated with the sparsely granulated phenotype than tumors showing absent/weak ARNT expression (scores 0–1) (60% vs. 17.6%, *p* = 0.002). Patients with tumors showing moderate/strong ARNT expression were younger at diagnosis than those with absent/weak ARNT expression (39 (32.5-50.25) vs. 46 (41–54) years, *p* = 0.044). Moderate/strong ARNT expression was also associated with lower GH immunohistochemical scores (*p* = 0.008), whereas baseline circulating GH levels did not differ significantly between groups (*p* = 0.273)

No statistically significant associations were identified between ARNT expression and sex, tumor size, invasiveness, sphenoid sinus invasion, suprasellar extension, optochiasmatic involvement, IGF1 × ULN, Ki-67 labeling index, AIP immunohistochemical expression or AHR immunohistochemical expression

In an exploratory multivariable logistic regression model, moderate/strong ARNT expression remained significantly associated with the sparsely granulated phenotype after adjustment for age at diagnosis and sex (adjusted OR 6.84, 95% CI 2.05–22.84; p = 0.002), whereas neither age at diagnosis nor sex was significantly associated with the granulation pattern (Table 5)

Importantly, the association was also preserved when ARNT expression was analyzed using the four-tier ordinal ARNT score (0–3), with each one-point increase in ARNT score associated with higher odds of the SG phenotype after adjustment for age and sex (adjusted OR 2.97, 95% CI 1.38–6.41; *p* = 0.005)

Sensitivity analyses were performed after excluding the two patients who had received preoperative first-generation somatostatin receptor ligand therapy (*n* = 69). The main findings remained unchanged: moderate/strong ARNT expression remained significantly associated with the SG phenotype after adjustment for age and sex (adjusted OR 6.50, 95% CI 1.94–21.74; *p* = 0.002). Similarly, when ARNT was analyzed as an ordinal variable, each one-point increase in ARNT score remained associated with higher odds of the SG phenotype (adjusted OR 2.87, 95% CI 1.33–6.18; *p* = 0.007)

Pairwise Spearman correlation analysis did not identify significant correlations between AIP, AHR and ARNT immunohistochemical expression in the overall cohort (AIP vs AHR: ρ = 0.183, p = 0.126; AIP vs ARNT: ρ = -0.004, p = 0.971; AHR vs ARNT: ρ = 0.128, p = 0.287). Similar findings were observed after stratification according to granulation pattern.

**Fig. 1:**
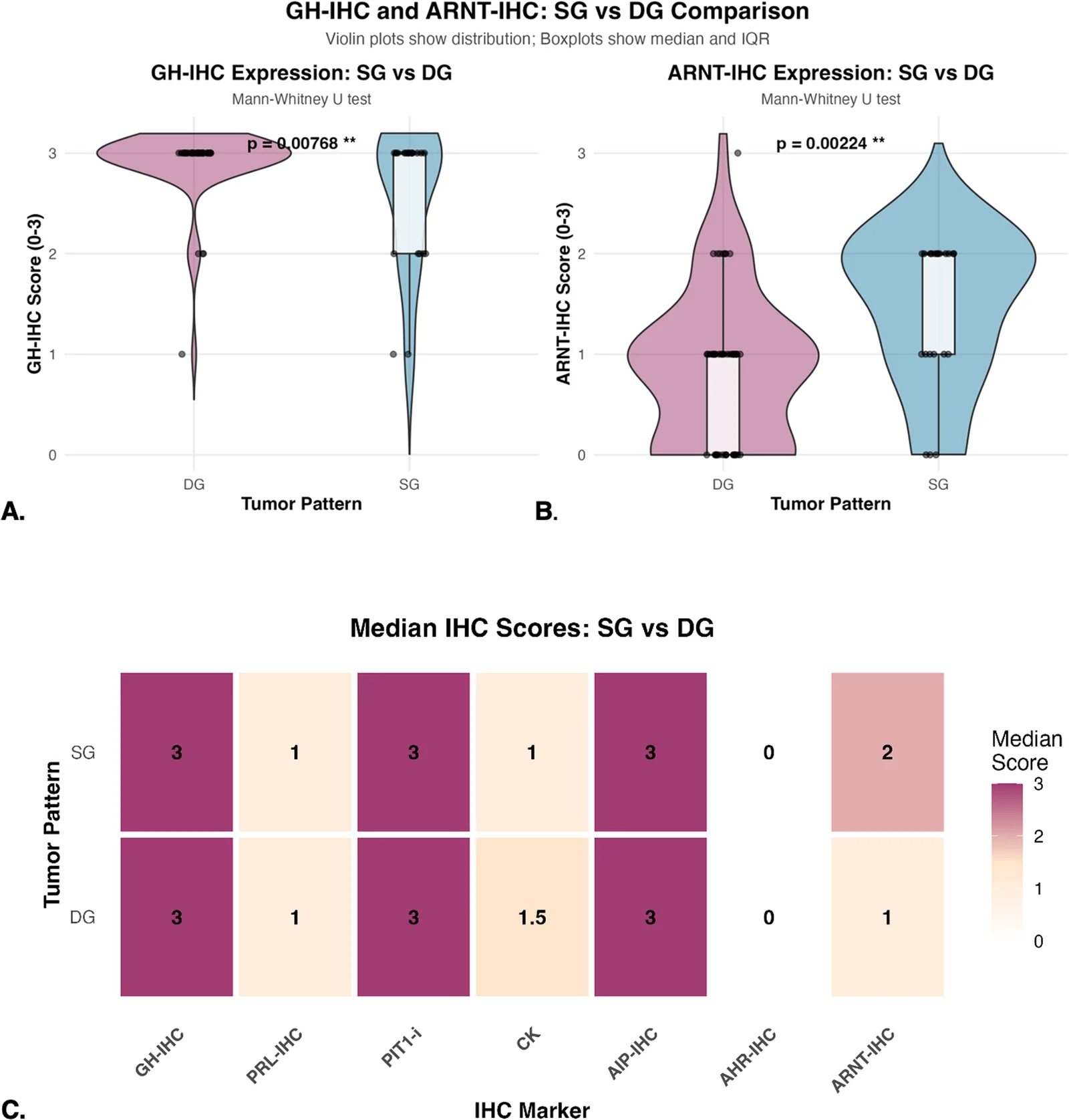
Immunohistochemical expression according to granulation pattern in somatotroph pituitary adenomas. (**A**) Distribution of GH immunohistochemical (GH-IHC) scores in densely granulated (DG) and sparsely granulated (SG) somatotroph adenomas. Individual observations are shown together with violin plots and boxplots. Although the median GH-IHC score was 3 in both groups, the overall distribution of ordinal scores differed significantly between DG and SG tumors (Mann-Whitney U test, *p* = 0.00768) (**B**) Distribution of ARNT immunohistochemical (ARNT-IHC) scores according to granulation pattern. ARNT scores were significantly higher in SG than in DG tumors (Mann-Whitney U test, *p* = 0.00224). Individual observations are shown together with violin plots and boxplots (**C**) Heatmap showing median immunohistochemical scores for GH, PRL, PIT1, cytokeratin AE1/AE3 (CK), AIP, AHR, and ARNT according to DG and SG granulation pattern

**Fig. 2:**
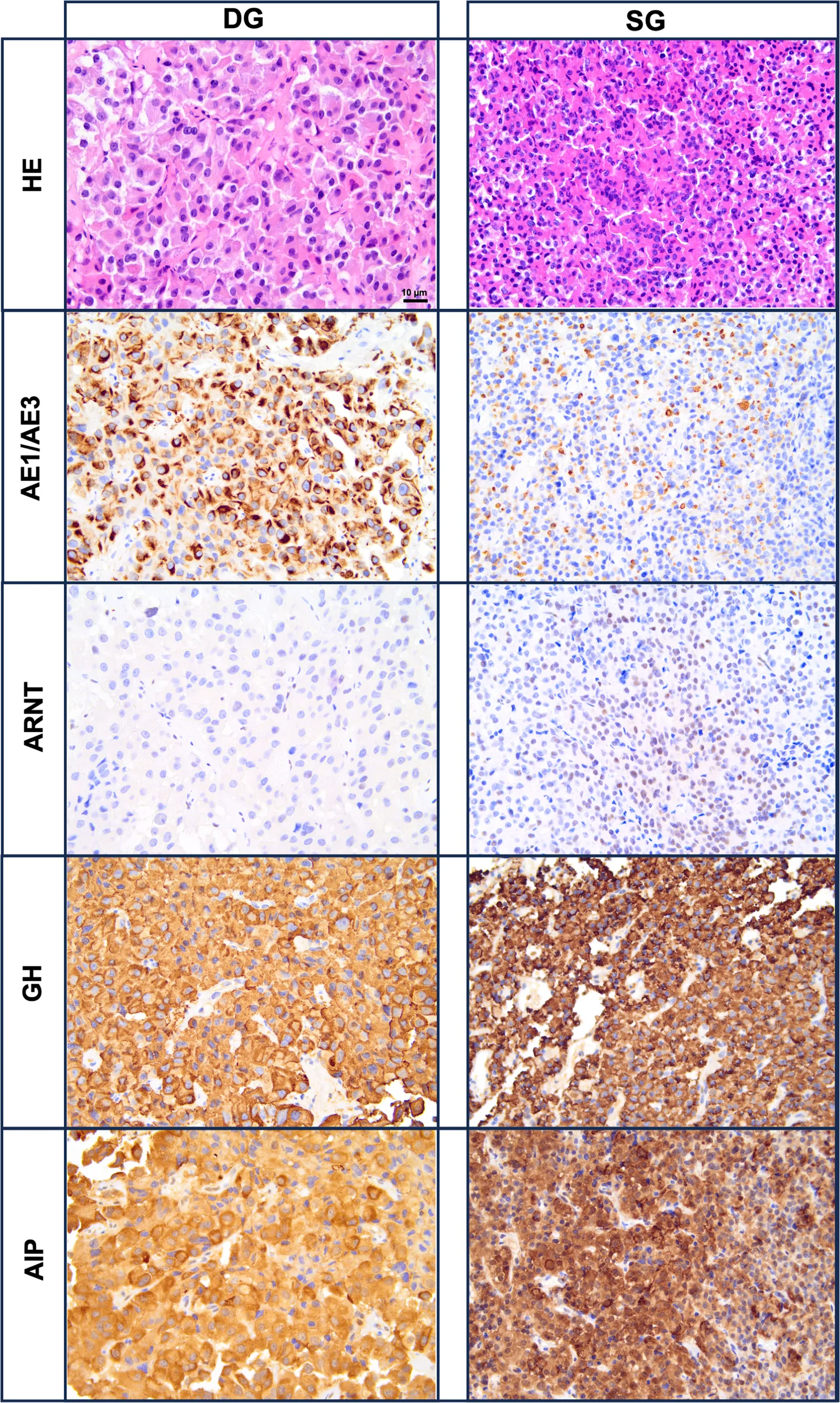
Representative histopathological and immunohistochemical findings in densely granulated (DG) and sparsely granulated (SG) somatotroph pituitary adenomas. Representative H&E, AE1/AE3, ARNT, GH, and AIP staining is shown for both granulation patterns. The DG adenoma demonstrates diffuse cytoplasmic AE1/AE3 staining, whereas the SG adenoma shows the characteristic paranuclear fibrous body pattern. Representative ARNT, GH, and AIP immunoreactivity is illustrated for both tumors. Images were acquired using a ×40 objective. Scale bar, 10 μm

The distributions of GH and ARNT immunohistochemical scores according to granulation pattern, together with the median immunohistochemical profile of the evaluated markers, are illustrated in Figure 1. Representative H&E, AE1/AE3, ARNT, GH and AIP staining in densely and sparsely granulated somatotroph pituitary adenomas is shown in Figure 2.

**Table 4:** Association between ARNT expression and clinicopathological characteristics

Variable	ARNT absent/weak (0–1)	ARNT moderate/strong (2–3)	*p* value
Sparsely granulated tumors, n (%)	9 (17.6)	12 (60)	0.002
Age at diagnosis, years	46 (41–54)	39 (32.5–50.3)	0.044
GH-IHC score	3 (3–3)	3 (2–3)	0.008
Baseline GH, ng/mL	9.9 (4.6–19.9)	7 (3.2–12.4)	0.273
IGF1 xULN	3.4 (2.7–4.3)	3.09 (2.5–4.2)	0.574
Female sex, n (%)	34 (66.7)	11 (55)	0.417
Macroadenoma, n (%)	46 (90.2)	18 (90)	1.000
Invasive tumors, n (%)	19 (37.3)	8 (40)	1.000
Sphenoid sinus invasion, n (%)	4 (7.8)	3 (15)	0.394
Suprasellar extension, n (%)	17 (33.3)	9 (45)	0.417
Optochiasmatic involvement, n (%)	13 (25.5)	6 (30)	0.769
Ki-67 < 3%, n (%)	40 (78.4)	12 (60)	0.141

Data are presented as median (interquartile range) or number (%)

**Table 5:** Multivariable logistic regression evaluating the association between moderate/strong ARNT expression and the sparsely granulated phenotype

Variable	Adjusted OR	95% CI	*p* value
Moderate/strong vs. absent/weak ARNT expression (scores 2–3 vs. 0–1)	6.84	2.05–22.84	0.002
Age at diagnosis	0.98	0.93–1.02	0.294
Female sex	1.82	0.53–6.27	0.342

Adjusted odds ratios (ORs) with 95% confidence intervals (CIs) derived from a multivariable logistic regression model with the sparsely granulated phenotype as the dependent variable. The model was adjusted for age at diagnosis and sex

Clinical outcome data were additionally evaluated to explore the potential clinical relevance of ARNT expression. Postoperative biochemical remission occurred in 8/51 (15.7%) patients with absent/weak ARNT expression and 3/20 (15.0%) patients with moderate/strong expression (*p* = 1.000). No significant associations were observed between ARNT expression and residual tumor (78.4% vs. 85.0%; *p* = 0.743) or the need for postoperative medical treatment (66.7% vs. 70.0%; *p* = 1.000). Among patients with persistent disease after surgery who subsequently received medical treatment (*n* = 48), biochemical disease control at the last evaluation was achieved in 22/34 (64.7%) patients with absent/weak ARNT expression and 9/14 (64.3%) patients with moderate/strong expression (*p* = 1.000). All 48 patients receiving postoperative medical therapy had been treated with a first-generation somatostatin receptor ligand. Inadequate biochemical control during first-generation SRL therapy was observed in 9/34 (26.5%) patients with absent/weak ARNT expression and 5/14 (35.7%) patients with moderate/strong expression, with no significant association with ARNT expression (*p* = 0.728)

## Discussion

Our study demonstrates that ARNT expression is significantly higher in SG than in DG somatotroph pituitary adenomas. This was the central clinicopathological finding of the present study. Moderate/strong ARNT expression remained significantly associated with the SG phenotype after adjustment for age and sex, and this association was preserved when ARNT was analyzed using the four-tier ordinal ARNT score (0–3), supporting the robustness of the finding. In univariable analyses, tumors with moderate/strong ARNT expression were also associated with younger age at diagnosis and lower GH immunohistochemical expression, whereas no significant associations were observed with Ki-67, AIP, or AHR expression. The associations with younger age and lower GH immunoreactivity should be interpreted in the context of the strong relationship between ARNT expression and the SG phenotype, as these are recognized characteristics of SG somatotroph adenomas, rather than as independent associations of ARNT with age or GH expression [4–7, 20].

This clinicopathological association is relevant because granulation pattern is a well-established histopathological feature of somatotroph adenomas, associated with differences in morphology, MRI appearance, treatment response and, in some series, invasiveness or surgical outcome [4–7, 20–22]. Contemporary reviews and consensus recommendations emphasize the importance of pathological tumor characteristics in the individualized management of acromegaly and in predicting treatment outcomes [18, 23].

The biological plausibility of this association may be considered in the context of ARNT-dependent signaling. ARNT, also known as HIF-1β, is a basic helix-loop-helix PAS family protein that serves as a dimerization partner for both ligand-activated AHR and hypoxia-inducible factor α-subunits, thereby participating in AHR- and hypoxia-related transcriptional responses [11, 21–22, 24]. Experimental evidence indicates that ARNT expression may itself be influenced by hypoxic conditions and that crosstalk between AHR and HIF signaling can occur at the level of ARNT [22, 24]. However, the present immunohistochemical data do not establish activation of these pathways or a causal relationship between ARNT expression and the SG phenotype.

No significant association was observed between ARNT and either AIP or AHR immunohistochemical expression. However, AIP expression was predominantly strong, whereas AHR expression was predominantly absent or weak, resulting in limited variability in both markers and reducing the ability to detect associations with ARNT. The different immunohistochemical distributions of AHR and ARNT are not necessarily unexpected despite their functional interaction, as ARNT also participates in other transcriptional pathways, including HIF signaling, and immunohistochemical protein abundance does not directly reflect AHR-ARNT heterodimer formation or pathway activation. Therefore, the absence of an association between AHR and ARNT expression should not be interpreted as evidence against functional interaction between these proteins.

Previous work has established the relevance of both AIP and AHR to pituitary tumor biology. AHR signaling appears downregulated in pituitary adenomas overall, AHR can exert tumor-suppressive effects through Rb/E2F-related cell-cycle regulation, and AIP loss contributes to pituitary tumorigenesis through altered cAMP/Gαi/PKA signaling and related pathways [10, 21, 25, 26]. In somatotroph tumors, reduced AIP expression has been associated in some cohorts with larger tumor size, SG morphology and poorer response to octreotide, whereas other studies reported associations with tumor size and subtype but not with treatment response [27–29]. Importantly, 69 of 71 patients (97.2%) in our cohort were treatment-naive before surgery, limiting the potential confounding effect of preoperative somatostatin receptor ligand exposure on AIP immunoexpression. Against this background, the lack of a detectable association between ARNT expression and AIP or AHR immunoexpression in our cohort does not allow firm conclusions regarding the functional relationship between these proteins. Experimental evidence showing that pituitary Aip-driven tumorigenesis is not fully recapitulated by Ahr loss or Pit1-lineage Arnt deletion nevertheless indicates that AIP-associated somatotroph tumorigenesis involves mechanisms beyond deficient AHR or ARNT activity alone [30]. Therefore, while our immunohistochemical findings do not establish whether ARNT expression is functionally linked to AIP/AHR signaling, they support further investigation of ARNT within the broader molecular context of somatotroph tumor heterogeneity.

The association between ARNT and the SG phenotype also fits emerging omics-based views of somatotroph tumor heterogeneity. Transcriptomic and proteogenomic studies indicate that SG tumors are molecularly distinct from DG tumors and are characterized by lower GH1 expression together with enrichment of signaling pathways related to inflammatory responses, JAK-STAT activity and cellular plasticity [31–36]. Although these findings do not demonstrate a causal role for ARNT, they provide a plausible biological context for our observations. These findings may indicate that ARNT participates in, or accompanies, a less terminally differentiated PIT1-lineage tumor state characterized by reduced GH production and storage relative to DG tumors while retaining lineage commitment. This interpretation is consistent with the lower GH immunoreactivity observed in tumors with greater ARNT expression in our cohort, although direct regulation of PIT1-lineage differentiation by ARNT remains to be established.

Our Ki-67 results also deserve careful consideration. ARNT expression was not significantly associated with the Ki-67 labeling index, providing no evidence for a relationship between ARNT expression and proliferative activity in this cohort. Granulation pattern and proliferative indices are only imperfectly related in somatotroph tumors, and biological behavior is influenced by additional genetic, epigenetic and microenvironmental factors that are not captured by morphology alone [2, 4, 7, 33, 36]. Consequently, a marker associated with differentiation state may still have biological value even in the absence of a direct relationship with Ki-67. The lack of a significant association between ARNT expression and Ki-67 suggests that the observed relationship with the SG phenotype is not simply paralleled by differences in this conventional proliferation marker.

Exploratory analyses of clinical outcomes did not identify significant associations between ARNT expression and postoperative biochemical remission, residual tumor, or the need for postoperative medical treatment. Furthermore, among patients with persistent disease who subsequently received medical therapy, ARNT expression was not associated with biochemical disease control at the last evaluation. Similarly, no significant association was observed between ARNT expression and biochemical control during first-generation SRL therapy. These findings are particularly relevant given the established relationship between granulation pattern and responsiveness to first-generation SRLs, with SG tumors generally showing a less favorable biochemical response than DG tumors [18]. In the present cohort, however, ARNT expression did not identify a clinically distinct subgroup in terms of the evaluated treatment outcomes.

The translational implications should therefore be stated cautiously. Although higher ARNT expression is strongly associated with the SG phenotype, our exploratory outcome analyses did not demonstrate additional prognostic or therapeutic information associated with ARNT expression. ARNT immunohistochemistry should not be considered a substitute for cytokeratin-based classification of DG and SG tumors, nor do the present findings support its use as an independent clinical biomarker. Whether ARNT may provide additional information in larger cohorts, in somatotroph tumors with intermediate or less clearly defined granulation patterns, or in conjunction with other molecular markers remains to be established [22, 24, 37, 38].

Several limitations should be considered when interpreting our findings. AIP, AHR, and ARNT expression was assessed using semi-quantitative immunohistochemical scoring, which is inherently subject to pre-analytical variation and observer-dependent assessment. Although staining was independently evaluated by two experienced pituitary pathologists and disagreements were resolved by consensus, the granulation pattern had been established before AIP, AHR, and ARNT scoring; therefore, the pathologists were not blinded to DG/SG classification. This may have introduced observer bias, particularly for the semi-quantitative assessment of ARNT expression. In addition, although the cohort included 71 tumors, only 21 were classified as SG and 20 showed moderate/strong ARNT expression, which should be considered when interpreting subgroup analyses and the confidence intervals of the multivariable estimates. Immunoreactivity reflects protein abundance rather than functional activation, intracellular trafficking, dimerization status, or transcriptional activity. This is particularly relevant for ARNT because its biological activity depends on binding partners, cellular localization, and oxygen-dependent signaling context [10, 21–22, 24]. No functional experiments were performed; therefore, causality cannot be inferred. Finally, the study lacks transcriptomic, proteomic, or spatial data that could determine whether higher ARNT expression co-segregates with hypoxia-related signatures, inflammatory pathways, lower GH1 expression, or other SG-associated molecular programs identified in recent omics studies [31–36]. Although postoperative and treatment-related outcomes were retrospectively evaluated, these analyses were exploratory and were limited by the sample size and the absence of detailed longitudinal biochemical measurements. Therefore, larger prospective cohorts with standardized longitudinal assessment are required to determine whether ARNT has prognostic or therapeutic value beyond its association with granulation pattern.

Future studies should therefore pursue several complementary directions. Functional experiments using ARNT knock-down or overexpression models in somatotroph cells should evaluate effects on GH1/POU1F1 expression, GH secretion, keratin organization and downstream AHR/HIF signaling. Parallel translational studies should determine whether tumors with greater ARNT expression exhibit coordinated expression of HIF-related markers, AHR pathway activation or established SG-associated transcriptomic signatures. Larger multicenter cohorts incorporating digital pathology and long-term clinical follow-up will also be required to validate the present findings and to determine whether ARNT carries prognostic or therapeutic relevance beyond its association with granulation pattern.

In conclusion, increased ARNT expression is associated with the sparsely granulated phenotype in somatotroph adenomas. This association remained significant after adjustment for age and sex and when ARNT was analyzed using the four-tier ordinal ARNT score (0–3). ARNT expression was not significantly associated with Ki-67, AIP, or AHR expression, nor with the clinical outcomes evaluated in exploratory analyses. These findings identify ARNT as a feature associated with the SG phenotype, although the present data do not establish additional prognostic or therapeutic utility beyond granulation pattern.

### Statistical analysis

Statistical analysis was performed using IBM SPSS Statistics version 30.0 (IBM Corp., Armonk, NY, USA). Continuous variables were expressed as median and interquartile range (IQR), while categorical variables were reported as absolute numbers and percentages.

Comparisons between groups were performed using the Mann-Whitney U test for continuous variables and the chi-square test or Fisher’s exact test for categorical variables, as appropriate. ARNT expression was analyzed both as an ordinal variable (scores 0–3) and as a dichotomized variable (absent/weak expression: scores 0–1; moderate/strong expression: scores 2–3).

Pairwise correlations between AIP, AHR and ARNT immunohistochemical expression were evaluated using Spearman’s rank correlation coefficient (Spearman’s rho), both in the overall cohort and after stratification according to granulation pattern.

Following univariable analyses, an exploratory multivariable logistic regression model was constructed to assess whether the association between moderate/strong ARNT expression and the SG phenotype remained significant after adjustment for age at diagnosis and sex. Variables included in the multivariable model were selected a priori based on biological relevance. Adjusted odds ratios (ORs) with 95% confidence intervals (CIs) were calculated.

A two-sided p-value < 0.05 was considered statistically significant.

## Supplementary Information

Below is the link to the electronic supplementary material.

Supplementary Material 1

## Data Availability

The datasets generated and/or analyzed during the current study are not publicly available due to patient confidentiality and institutional data protection regulations but are available from the corresponding author on reasonable request, subject to approval by the institutional ethics committee where applicable.
