# Supplementary material for: Higher ARNT expression is associated with the sparsely granulated phenotype in somatotroph pituitary adenomas: An immunohistochemical study
Source: Pituitary. 2026 Oct 16;29(6):171. doi: 10.1007/s11102-026-01776-5 (PMC13675150; doi:10.1007/s11102-026-01776-5)
Supplement: Supplementary file 1 [file 11102_2026_1776_MOESM1_ESM.docx]

**Supplementary Figure S1 Representative positive and negative controls for AIP, AHR, and ARNT immunohistochemistry.** Positive controls consisted of normal adenohypophyseal tissue for AIP, lymphocytes within secondary lymphoid aggregates in liver tissue for AHR, and formalin-fixed, paraffin-embedded normal human lung tissue for ARNT. Negative controls were performed for each antibody by replacing the primary antibody with antibody diluent alone.
